# Poncirus Trifoliata (L.) Raf. Extract Inhibits the Development of Atopic Dermatitis-like Lesions in Human Keratinocytes and NC/Nga mice

**DOI:** 10.7150/ijms.34323

**Published:** 2019-08-06

**Authors:** Kyung-Jae Cha, Ayesha Kashif, Min Hwa Hong, Geunyeong Kim, Ji-Sook Lee, In Sik Kim

**Affiliations:** 1 Department of Senior Healthcare, BK21 Plus Program, Graduate School, Eulji University, Daejeon 34824;; 2 Department of Clinical Laboratory Science, Wonkwang Health Science University, Iksan, 54538;; 3 Department of Biomedical Laboratory Science, School of Medicine, Eulji University, Daejeon 34824, Republic of Korea.

**Keywords:** Atopic dermatitis, Poncirus trifoliata (L.) Raf., Anti-inflammatory effect, Filaggrin

## Abstract

This study investigated the anti-allergic effect of Poncirus trifoliata (L.) Raf. (PT) on human keratinocytic HaCaT cells *in vitro* and on 2,4‐dinitrochlorobenzene (DNCB)-induced atopic dermatitis-like lesions *in vivo*. The release of TARC, MCP-1, IL-6 and IL‐8 is increased by IFN-γ and TNF-α in HaCaT cells, and PT extract suppressed the increased production of TARC, MCP-1, IL-6, and IL‐8. PT extract recovered the expression of filaggrin decreased by IFN-γ and TNF-α. *in vivo* experiment, PT administration decreased the skin severity score, thickening of the epidermis, movement of inflammatory cells into the dermis, and serum IgE level as compared to DNCB treatment. Moreover, the decrease of filaggrin and loricrin induced by DNCB treatment was recovered by PT administration. The levels of IL-4, IL-5, IL-13 and eotaxin in mouse splenocytes increased after treatment with concanavalin A, and the secretions of IL-4, IL-5, IL-13 and eotaxin were lower in the PT-treated group than in the DNCB group. These findings may indicate that PT is useful in drug development for the treatment of AD.

## Introduction

Atopic dermatitis (AD; eczema) is a type of hypersensitivity of the skin. AD primarily occurs in infant and children and is involved in excess immune responses to allergens, immune deviation, barrier dysfunction and genetic abnormality 1-3. The level of serum immunoglobulin E (IgE) increases in patients with AD and includes antibodies to a variety of food and allergens 4, 5. AD is characterized by an increase in inflammatory cells and cytokines, and by a decrease in skin barrier proteins such as filaggrin 6-8. Filaggrin is an important protein expressed in keratinocytes and is related to the maintenance of skin barrier role 9, 10. Impaired filaggrin can contribute to allergic sensitization, enhance inflammatory responses accompanied by erythema, itchiness, and scratching of the skin, and finally result in development and aggravation of AD.

Poncirus trifoliata (L.) Raf. (PT) is used as an herb in Korea for the treatment of gastrointestinal disorders 11. It has also been reported in anti-oxidant, anti-bacterial, and anti-allergic activities 12-16.

In the present study, we examined the suppressive effect of PT on cytokine secretion and the expression of skin barrier proteins such as filaggrin, loricrin, and involucrin *in vitro*. In addition, we investigated the effect of PT on attenuation of AD development in AD‑like NC/Nga mice *in vivo*.

## Materials and methods

### Preparation of PT extract

Whole PT plants (30g) were dried and incubated with DMSO for 24 h at room temperature. The complete PT extracts were used in this study. Voucher specimens No. 032-088) were stored at the herbaria of the Department of the Herbal Pharmaceutical Development, Korea Institute of Oriental Medicine, Daejeon, Korea.

### Cell culture

HaCaT cells were cultured in Iscove's medium and DMEM supplemented with 10% heat‑inactivated fetal bovine serum (FBS), penicillin (100 U/mL), and streptomycin (100 μg/mL) (Gibco‑BRL, Grand Island, NY, USA). The cultured cells were maintained at 5% CO_2_ incubator. Cell viability was assayed based on the conversion of MTT by using a cell proliferation kit (Roche Korea, Seoul, Korea).

### Enzyme-linked immunosorbent assay

After pretreatment with PT extract, HaCaT cells were treated with IFN‑γ and TNF‑α. Splenocytes were pretreated in the absence or presence of PT and then stimulated with 1 μg/mL concanavalin A (Sigma-Aldrich Korea, Seoul, Korea) for 24 h and 48 h. Cell supernatants were collected and the concentrations of TARC, IL-6, IL‑8, MCP‑1, IL‑4, IL‑5, IL‑13, and eotaxin were measured in the supernatant by sandwich ELISA (BD Biosciences, San Jose, CA, USA and R&D Systems). The concentrations of alanine aminotransferase (ALT) and aspartate aminotransferase (AST) in the serum of NC/Nga mice were measured by ALT and AST assay kits (Asan Pharm, Seoul, Korea).

### Western blotting

Following treatment with stimulatorα, HaCaT cells were harvested and lysed in lysis buffer. Samples were separated by performing 10% SDS-PAGE and then transferred to nitrocellulose membrane. Blots were incubated with antibodies against filaggrin, phospho-JNK (Santa Cruz Biotechnology, Santa Cruz, CA, USA), involucrin, or loricrin (Proteintech, Rosemont, IL, USA). After incubation, the membrane was developed by using an enhanced chemiluminescence detection system (Amersham Pharmacia Biotech, Piscataway, NJ, USA).

### Atopic dermatitis induction and PT treatment in NC/Nga mice

* Female 5*-week‐old NC/Nga mice (25 ± 2 g) (SLC Japan, Shizuoka, Japan) were used in this experiment. They were housed in an air‐conditioned animal experiment room with a room temperature and a 50 ± 10% humidity. Before AD induction, the dorsal hair of NC/Nga mice was shaved off. There was not any sign of skin damage. AD was induced by stimulation with 2,4‐dinitrochlorobenzene (DNCB, Sigma‑Aldrich Korea). A 1% DNCB solution (0.15 mL) dissolved in an acetone-olive oil mixture (acetone:olive oil = 3:1) was applied to the shaved dorsal skin area. After this initial sensitization treatment, the mice were dorsally treated with 0.3% DNCB at 1 week intervals for 5 weeks. The NC/Nga mice were classified into four groups; untreated, control, PT, and dexamethasone (DEX) groups. The control, PT, and DEX groups were dorsally treated with 1% DNCB and thereafter were dorsally administered with 0.3% DNCB for 12 weeks. The control, PT, and DEX groups had phosphate-buffered saline (PBS), PT extract (100, 200, 500 μg /kg), and DEX (5 mg/kg), respectively, applied to the same area of dorsal skin for 7 weeks after sensitization with 0.3% DNCB. The untreated group was treated with PBS. The severity of dermatitis was assessed macroscopically in a blinded fashion according to our previous paper 6. Experimental procedures were approved by the Institutional animal care and use committee, Eulji University (Approval number: EUIACUC- 15-10).

### Histological analysis

After sacrificing the mice, the dorsal skin was separated and fixed in Carnoy's solution, embedded in paraffin (Sigma‑Aldrich Korea) and sectioned. The tissue sections were then stained with hematoxylin‑eosin solution or alcian blue (Sigma-Aldrich Korea). Finally, the sections were examined by using light microscopy (Leica Microsystems, Wetzlar, Germany) for histological evaluation. For immunohistochemical staining, we performed on 4 μm-thick paraffin sections with a automated tissue staining system of Ventana Medical Systems Inc. (TuPTon, AZ, USA). The sections were placed on SuperfrostPlus microscope slides (Fisher Scientific, Madison, WI, USA). An OptiView DAIHC Detection Kit (Ventana Medical Systems) was used as a 3,3′-diaminobenzidine (DAB) for detecting antibodies. Sections were deparaffinized with EZ Prep solution. CC1 standard (Tris/Borate/EDTA, pH 8.4) was used for antigen retrieval. Slides were incubated with anti-filaggrin, anti-involucrin or anti-loricrin antibody (Santa Cruz Biotechnology) after which they were incubated with OptiView HRP Multimer, HQ Universal Linker, and H_2_O_2_. After incubating with OptiView DAB and copper, they were counterstained and post-counterstained with hematoxylin-eosin and bluing reagent, respectively.

### Measurement of serum IgE

Blood was collected from the retro‐orbital plexus of the mice on the day of euthanizing. Serum was obtained by centrifugation and stored at -70°C until required. Total IgE levels in the serum were measured by using a sandwich ELISA kit (R&D Systems, Minneapolis, MN, USA).

### Splenocyte preparation

Mice were euthanized, and subsequently, their spleens were removed under aseptic conditions. Splenocytes were then isolated from the spleens after which the red blood cells were hemolyzed by using a red blood cell lysis solution (Sigma‑Aldrich). Splenocytes were seeded in a 24‑well plate at a density of 5×10^6^ cells/mL in RPMI‑1640 medium supplemented with 1% penicillin‑streptomycin and 10% FBS.

## Statistical analysis

Data are represented as a mean ± standard deviation (SD). Intergroup differences were evaluated by the Student's t-test within SPSS software (SPSS, Chicago, IL, USA). *P* < 0.05 was considered as a statistically significant difference.

## Results

### PT inhibits the cytokine release of HaCaT cells

**Figure 1:**
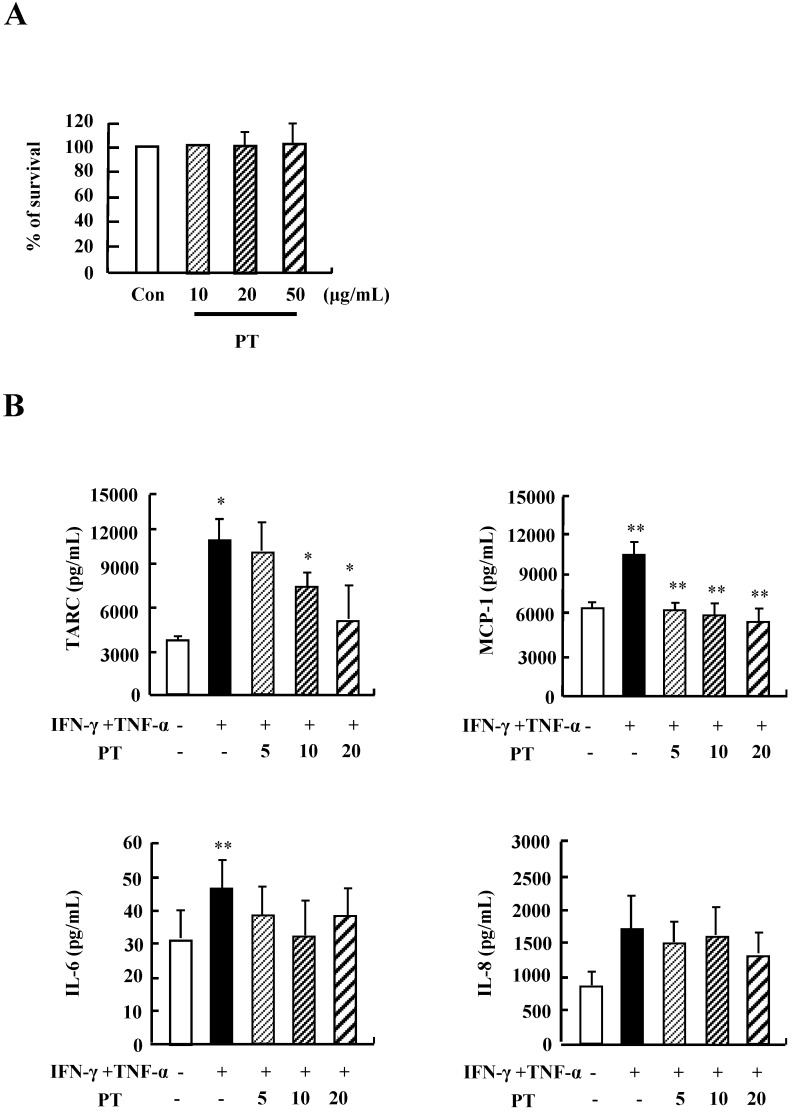
** PT inhibits the cytokine release of HaCaT cells. (A)** HaCaT cells were incubated in the absence (medium alone) or presence of PT extract at the indicated concentrations for 48 h. Survival rate was measured by performing MTT‑based viability assay. Data are presented as a mean ± SD of three independent experiments and expressed as a relative ratio to the absorbance of untreated cells, which was set at 100%d. **(B)** HaCaT cells were pretreated in the absence or presence of PT extract at the indicated concentrations. Cells were treated with 10 ng/mL IFN-γ and TNF-α for 24 h. The supernatant was collected and analyzed by using ELISA. Data are presented as the mean ± SD of three independent experiments with statistical significance as *P < 0.05 and **P < 0.01 between untreated and IFN-γ and TNF-α-treated groups or between the IFN-γ and TNF-α-treated group and the PT‑treated group.

We examined the optimal treatment concentration of PT extract in HaCaT cells. PT extract was not effective on survival rate of HaCaT cells after stimulation with PT extract at concentrations ranging from 10 ug/mL to 50 ug/mL for 48 h (Fig. 1A). Treatment with IFN-γ and TNF-α increased the secretion of TARC, MCP-1, IL-6 and IL-8 (Fig.1B). PT decreased the production of TARC, MCP-1, and IL-8 induced by IFN-γ and TNF-α stimulation. These results indicate that PT extract suppresses the secretion of inflammatory cytokines in HaCaT cells during an inflammatory response.

### PT extract reduces the decrease of filaggrin induced by IFN-γ and TNF-α

**Figure 2:**
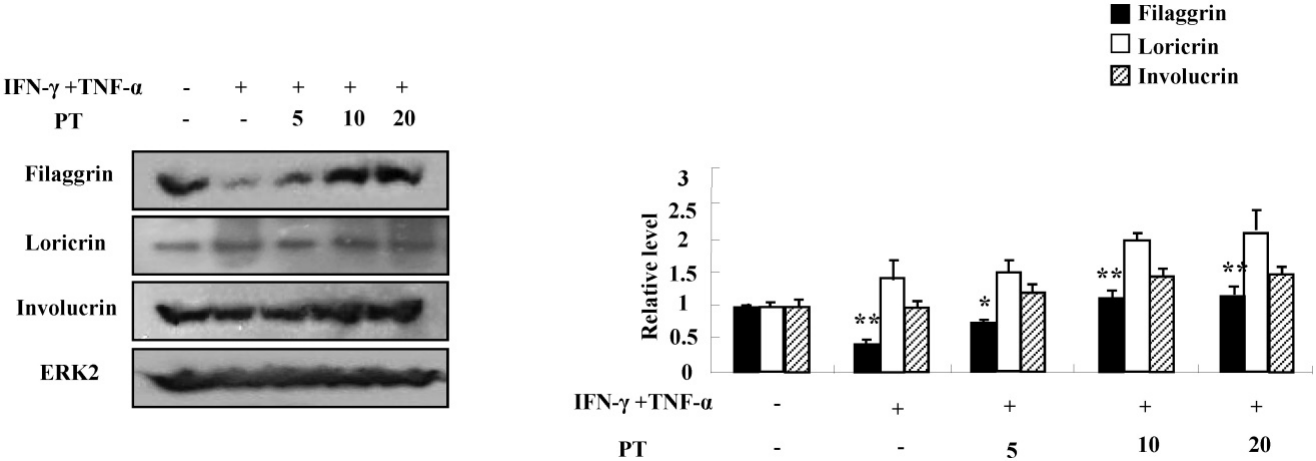
** PT recovers the decrease of filaggrin induced by IFN-γ and TNF-α.** HaCaT cells were preincubated in the absence and presence of PT at the indicated concentrations for 1 h. The cells were then incubated with 10 ng/mL IFN-γ and TNF-α for 48 h. The harvested cells were lysed, and filaggrin, loricrin and involucrin were analyzed by western blotting. Densitometric data are expressed as a mean ± SD and are presented relative to the negative control, which was set at 1 (right panel) with statistical significance as *P < 0.05 and **P < 0.01 between the untreated and IFN-γ and TNF-α-treated group or between the IFN-γ and TNF-α-treated group and the PT-treated group.

We next investigated whether PT extract alters the expressions of filaggrin, loricrin, and involucrin. IFN-γ and TNF-α suppressed the expression of filaggrin. The decreased expression was recovered by PT extract in a dose-dependent manner (Fig. 2). The expressions of loricrin and involucrin were increased or was not altered by IFN-γ and TNF-α, and PT extract increased the expressions of loricrin and involucrin. These results indicate that PT extract increases the expression of filaggrin under inflammatory processes that may result in a filaggrin decrease.

### PT extract decreases the aggravation of atopic-like skin lesion, histopathological features, and serum IgE in AD-induced mice

**Figure 3:**
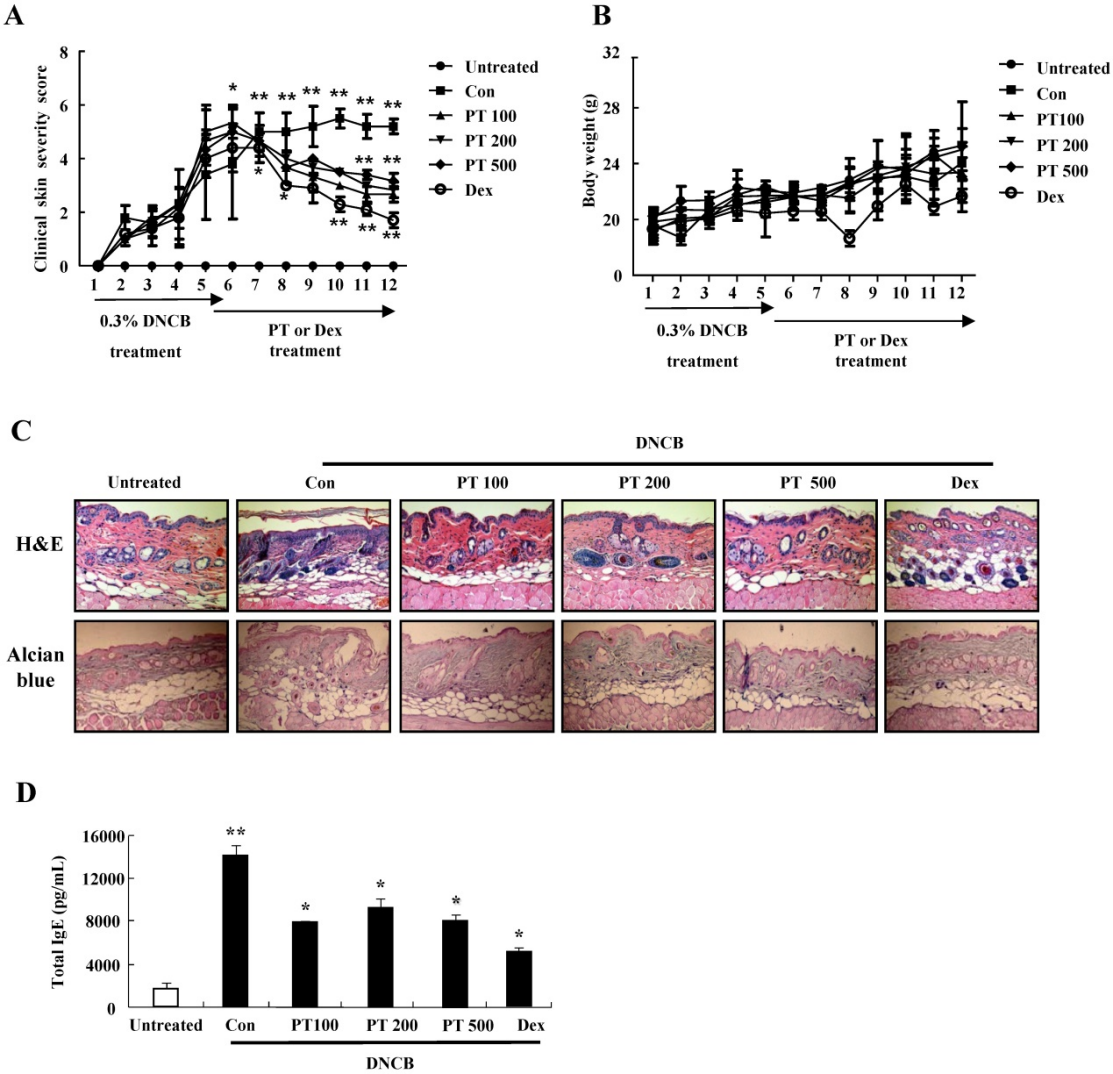
PT extract decreases the aggravation of atopic-like skin lesion, histopathological features, and serum IgE in DNCB-induced AD mice. The mice were divided into four groups: Untreated, control (Con), PT, and DEX. The control, PT, and DEX groups were dorsally administered with 1% DNCB and then dorsally treated with 0.3% DNCB. PT was administered orally at concentrations of 100, 200, and 500 µg/kg. DEX was administered orally at 5 mg/kg. **(A)** The severity of dermatitis was evaluted macroscopically in a blinded experiment. **(B)** Mouse mean body weight was measured by using an electric scale. Data are presented as a mean ± SD. **(C)** For histological analysis, the dorsal skin was fixed, embedded in paraffin, sectioned, stained with hematoxylin-eosin and alcian blue, and examined by using light microscopy (magnification, ×100).** (D)** Total serum IgE levels were measured by using sandwich ELISA kits. **(E)** The levels of AST and ALT were measured in the serum of NC/Nga mice by using the Reitman-Frankel method and ALT and AST assay kits. Data are presented as a mean ± SD with statistical significance as *P < 0.05 and **P < 0.01 between the untreated and control groups or between the control and PT-treated groups.

For evaluating the suppressive effect of PT in the pathogenesis of AD, we performed the clinical, histological, and serological analyses. NC/Nga mice were administered with DNCB for 5 weeks and thereafter PT extract was treated to the mice for 7 weeks. PT administration recovered the increase of a skin symptom severity score due to DNCB as compared to the control group, and the score of the PT-treated group was comparable to that of the DEX-treated group (Fig. 3A). The body weight of the PT-treated group was similar to that of the control group (Fig. 3B). Histological evaluation displayed hypertrophy, hyperkeratosis of the epidermis and infiltration of inflammatory cells in the control group (Fig. 3C). However, administration of PT extract relieved the histopathological alteration in a fashion comparable to the dexamethasone group. The level of serum IgE was higher in the control group than in the untreated group, while PT treatment blocked the increased IgE concentration in serum (Fig. 3D). Moreover, the serum AST and ALT in the PT-treated group were similar to those in the untreated group (Fig. 3E).

### PT extract enhances the expression of filaggrin in AD-induced mice

**Figure 4:**
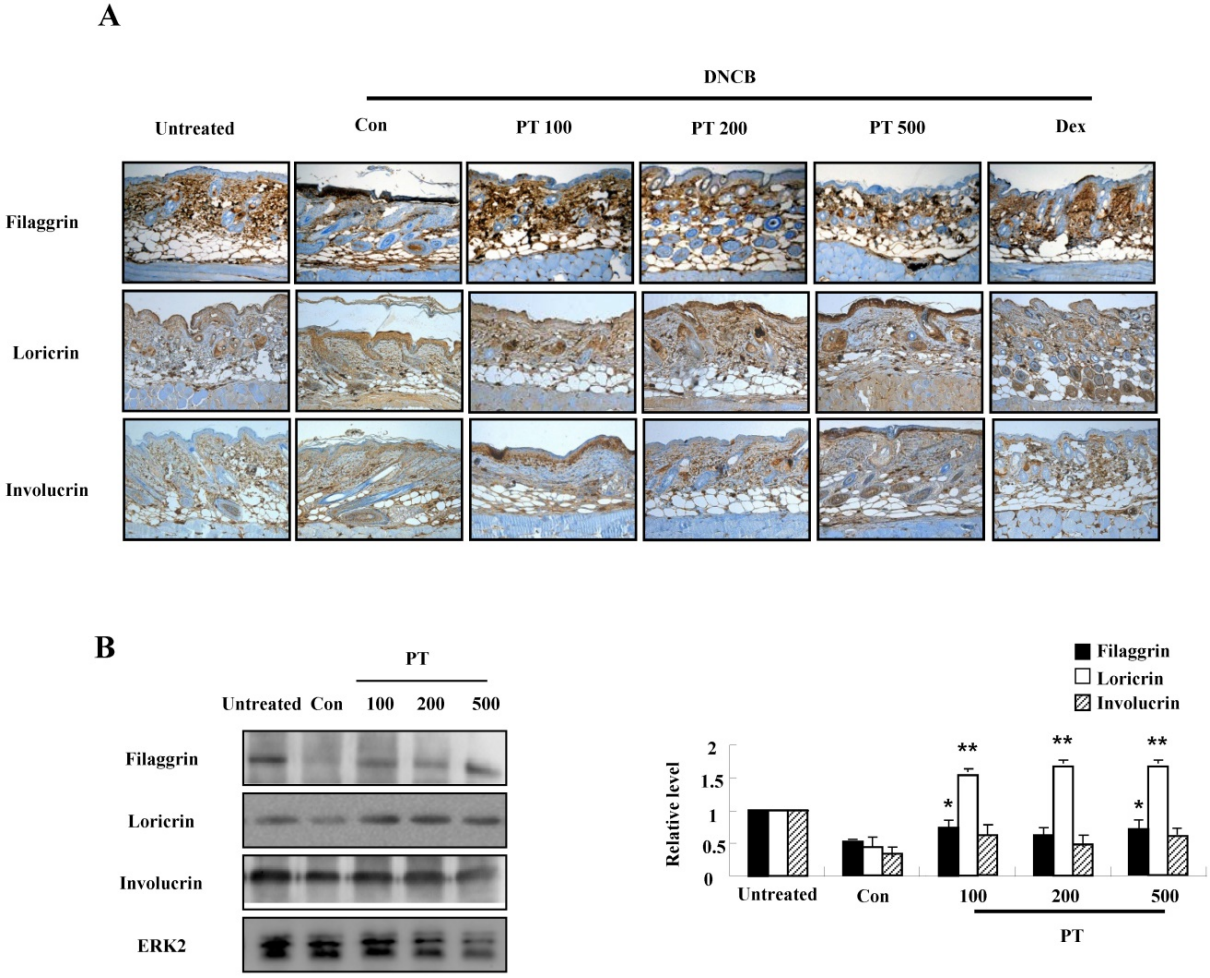
PT extract enhances the expression of filaggrin in skin of NC/Nga mice. **(A)** For filaggrin analysis, skin sections were fixed, embedded in paraffin, and stained with immunohistochemical stains. The samples were examined by using light microscopy (magnification, ×100). **(B)** Filaggrin, loricrin, and involucrin as well as phospho-JNK, in the dorsal skin were analyzed by western blotting. Densitometric data are expressed as means ± SD and are presented relative to the negative control, which was set at 1 (right panels of B). *P < 0.05 and **P < 0.01 indicate a statistical significance between the untreated and control groups or between the control and PT-treated groups.

To evaluate the effect of PT extract on filaggrin in AD-induced mice, we performed both immunohistochemical staining and western blotting. Filaggrin expression in epidermis was more intense in the PT-treated group than in the control group (Fig. 4A). In experiments using western blotting, the expressions of filaggrin, loricrin, and involucrin decreased after DNCB administration, and they were recovered in the PT-treated group (Fig. 4B).

### PT extract inhibits the secretion of IL-4, IL-5, IL-13, and eotaxin in mouse splenocytes

**Figure 5:**
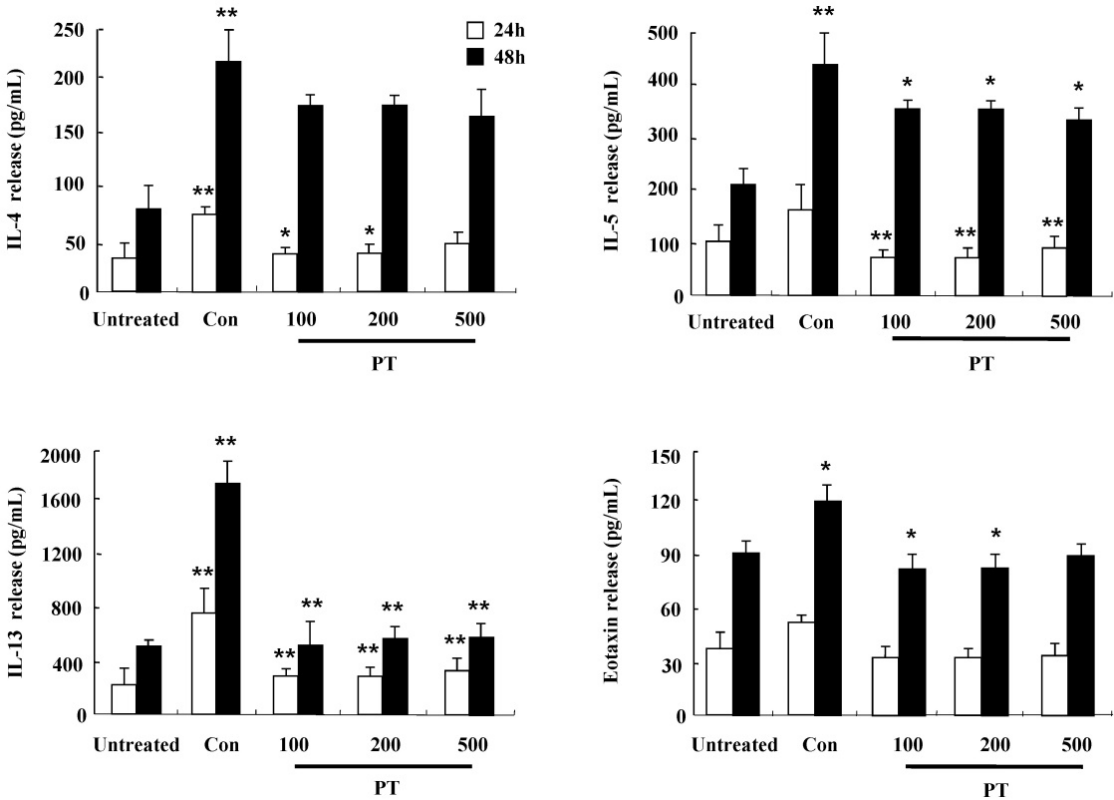
PT extract inhibits the secretion of IL-4, IL-5, IL-13, and eotaxin in mouse splenocytes. Splenocytes were isolated from NC/Nga mice of the untreated, control (Con), and PT groups. Subsequently, the cells were treated with 1 μg/mL concanavalin A for 24 h and 48 h. Supernatants were collected and analyzed by ELISA. Data are presented as means ± SD with statistical significance as *P < 0.05 and **P < 0.01 between the untreated and control groups or between the control and PT-treated groups.

To investigate the anti-inflammatory effect of PT in DNCB-induced mice, splenocytes were isolated from mouse spleen at 12 weeks after the first DNCB sensitization. After the stimulation with concanavalin A for 24 h and 48 h, the release of cytokines such as IL-4, IL-5, IL-13, and eotaxin increased in splenocytes of the control group, but the increased cytokines was diminished in splenocytes of the PT-treated group (Fig. 5). These results indicate that PT treatment affects the synthesis of cytokines and chemokines in the clinical state of AD.

## Discussion

PT has been known to reveal anti-allergic effect including inhibition of IgE production, histamine release, and IL-5 synthesis 12-14, 17. On that basis, this study was designed to examine the anti-inflammatory effect of PT on the pathogenesis of AD and the possibility of using PT extract in a therapeutic drug for the treatment of AD.

In the development and aggravation of AD, the regulation of cytokine secretion, particularly the Th1/Th2 cytokines and chemokines, is a key process [18). TARC, MCP-1, and IL-8 have been reported as survival factors and pathogenic inducers of AD 19. IL-6 is secreted from T lymphocytes, macrophages, and eosinophils, and it plays an essential role in the transition from an acute inflammatory state to a chronic inflammatory state 20. As shown in Fig. 1, PT extract decreased the expression of cytokines such as TARC, MCP-1, and IL-8 induced by IFN-γ and TNF-α in human keratinocytic HaCaT cells. In AD-like NC/Nga mice, the PT-treated group, after st with concanavalin A for 24 h and 48 h, represented lower production of Th2 cytokines such as IL-4, IL-5, and IL-13, and a chemokine, eotaxin, than the production levels in the control group (Fig. 5). Because IL-4, IL-5, and IL-13 is related to in increased IgE production in AD patients, PT may lower serum IgE level by suppressing the synthesis of Th2 cytokine (Fig. 3D) 21. PT also may inhibit histopathological features by lowering the level of eotaxin attracting eosinophils (Fig. 3). PT includes more than 50 phytochemicals such as poncirin, limonene, synephrine, hesperidin, neohesperidin, auraptene and imperatorin 11. 21-Methylmelianodiols are effective on inhibition of IL-5 production 17. Hesperidin ameliorates UV radiation-induced skin damage and Auraptene suppresses IL-4 production 22, 23. The alteration of cytokine expression in our results may be caused by these anti-inflammatory chemicals contained in PT extract.

Defects in skin barrier proteins are important for the development of AD. IFN-γ and TNF-α activate the mitogen-activated protein kinase-mediated mechanism, which regulates the expression of filaggrin 19, 24, 25. Our results demonstrate that IFN-γ and TNF-α suppressed filaggrin expression in HaCaT cells and PT increased filaggrin expression during AD state (Figs. 2 and 4). Loricrin and involucrin also are essential skin barrier proteins. PT enhanced the expressions of loricrin and involucrin, which have been decreased by DNCB treatment (Fig. 4). IL-4 decreases CBP binding to the involucrin transcription complex, which results in downregulation of involucrin expression 26. IL-13 plays an important role in downregulation of filaggrin, loricrin, and involucrin through STAT pathway 27. Increase of IL-4 and IL-13 in AD may be deeply implicated in downregulation of skin barrier proteins (Fig. 5). Further study is required to examine concise signal pathways activated by PT.

In AD-like NC/Nga mice, the DNCB-treated control group displayed increased clinical skin severity score, serum IgE level, and histopathological skin lesions (Figs. 4 and 5). The PT-treated group displayed a low skin symptom severity score, alleviation of histopathological features such as infiltration of inflammatory cells and epidermis hypertrophy, and a low serum IgE compared to those in the control group. These results are comparable to the effects of other herb extracts reported in other papers 7, 28.

In conclusion, we demonstrated that PT extract suppresses inflammatory cytokines and chemokines and that it alleviated the skin inflammation and defect of skin barrier proteins in AD-like NC/Nga mice. This work gives a new insight on the development of a therapeutic drug for the treatment of AD.

## Abbreviations

Dexamethasone: DEX
DNCB: 2,4‐dinitrochlorobenzene
PT: Poncirus Trifoliata (L.) Raf
